# Supplementary material for: CGRP causes anxiety via HP1γ–KLF11–MAOB pathway and dopamine in the dorsal hippocampus
Source: Commun Biol. 2024 Mar 19;7:322. doi: 10.1038/s42003-024-05937-9 (PMC10951359; doi:10.1038/s42003-024-05937-9)
Supplement: Supplementary file 2 — Supplementary Figs. [file 42003_2024_5937_MOESM2_ESM.pdf]

Supplementary Figure 1.

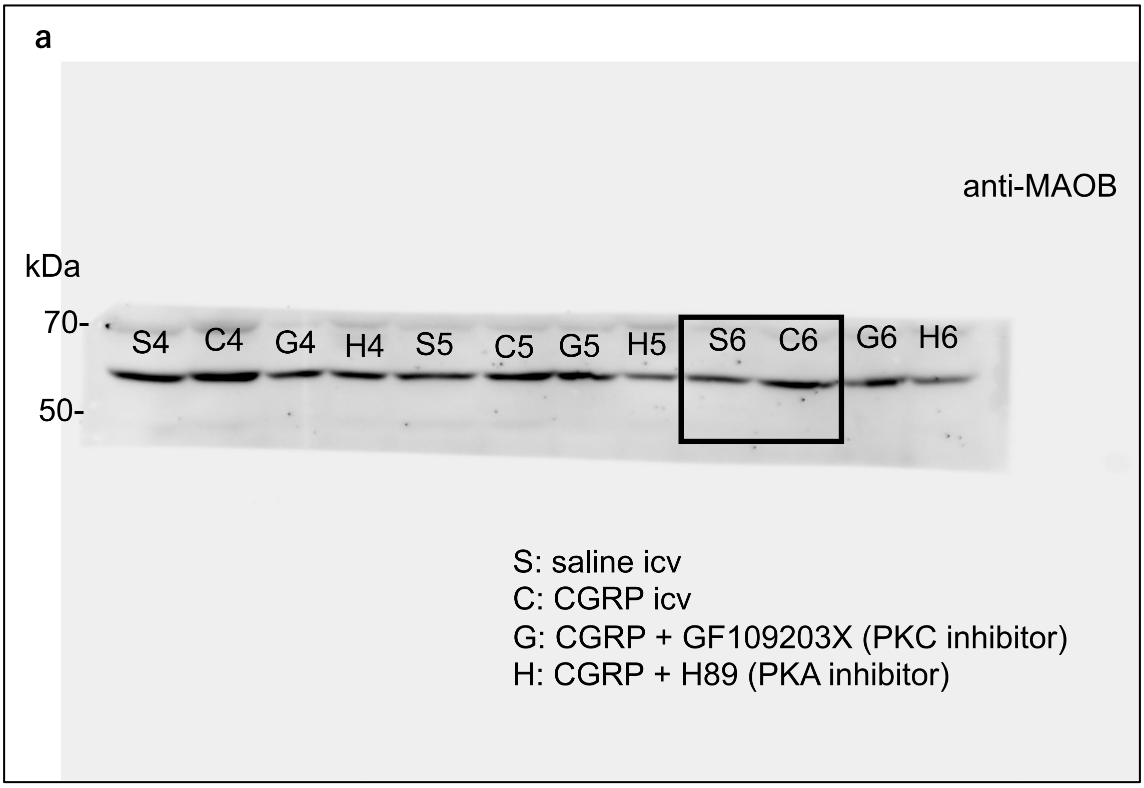


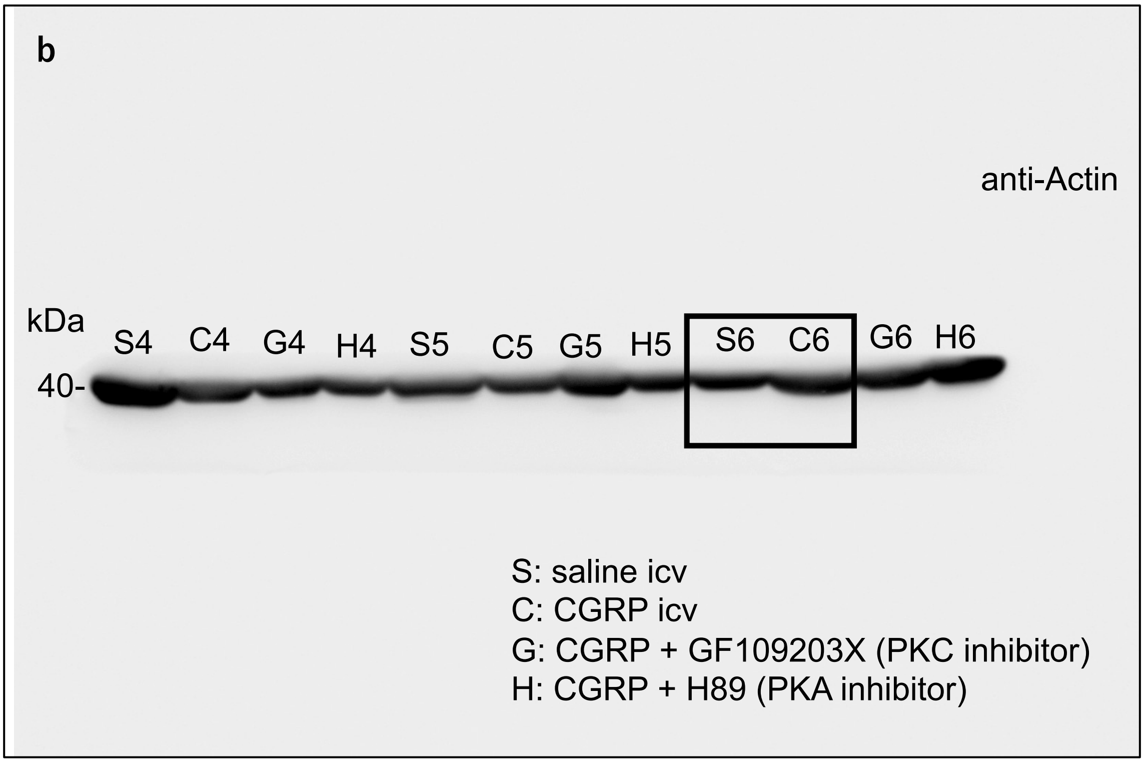


Supplementary Figure 1: Uncropped images of western blots.

(a) Western blot images for Figure 2c, anti-MAOB.

(b) Western blot images for Figure 2c, anti-Actin.

Supplementary Figure 2.

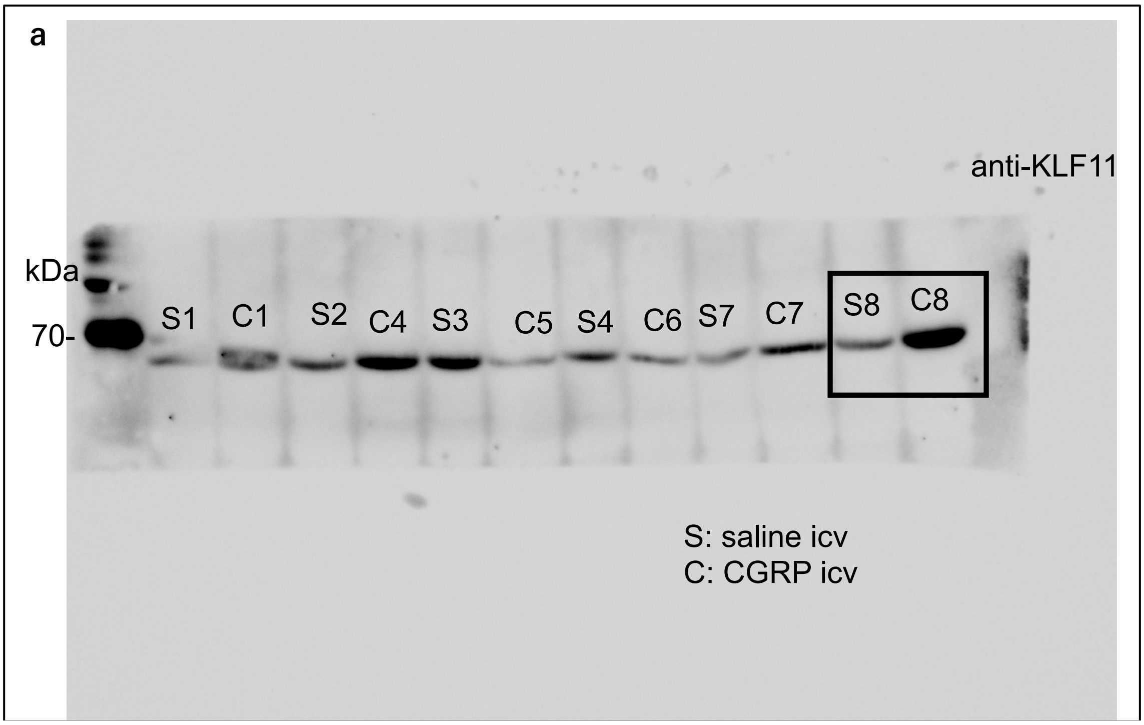


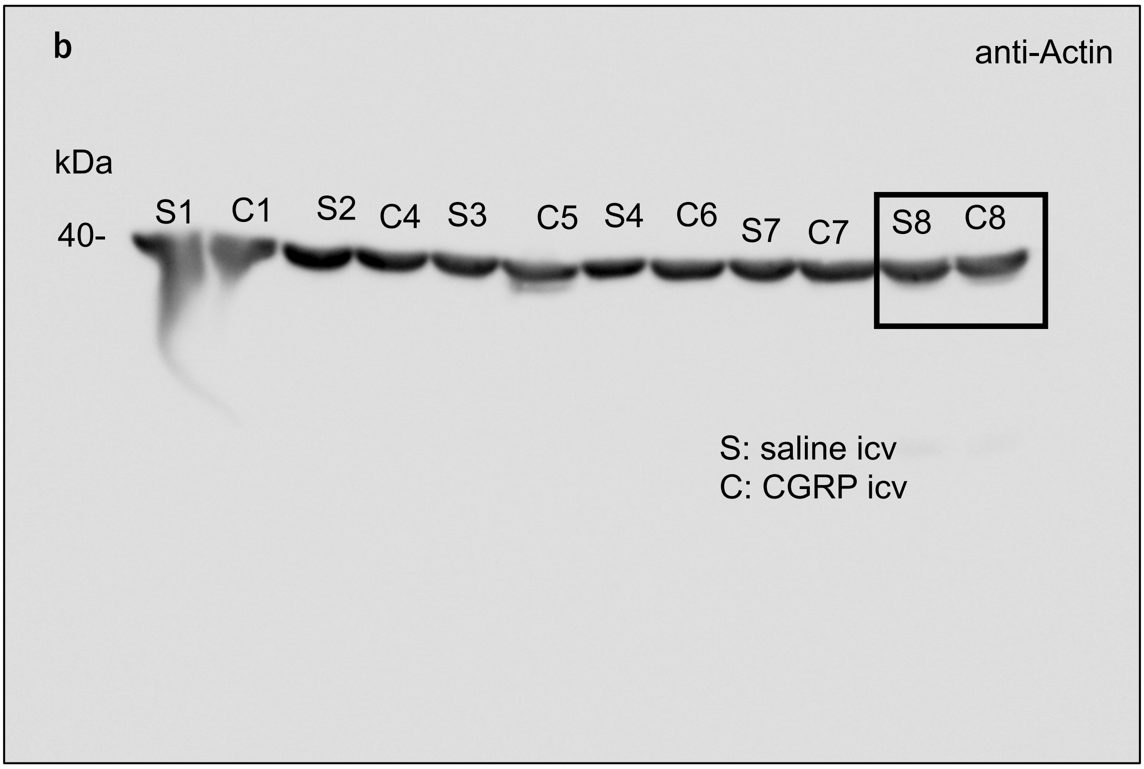

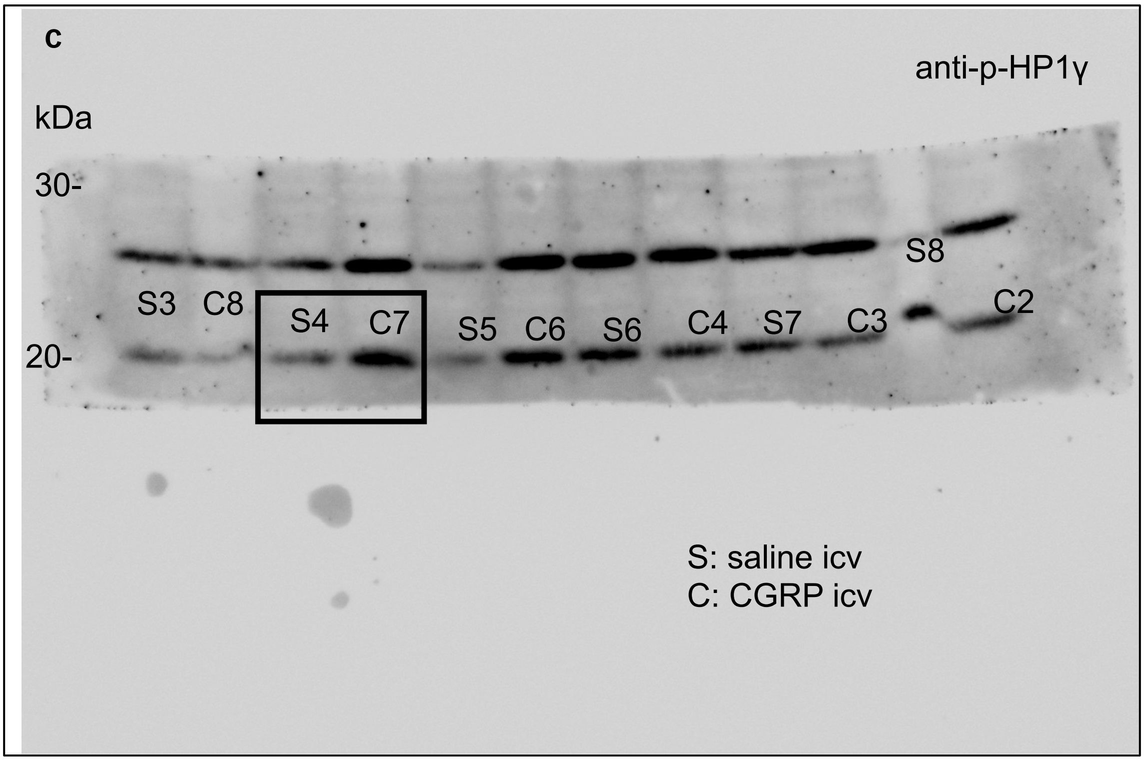


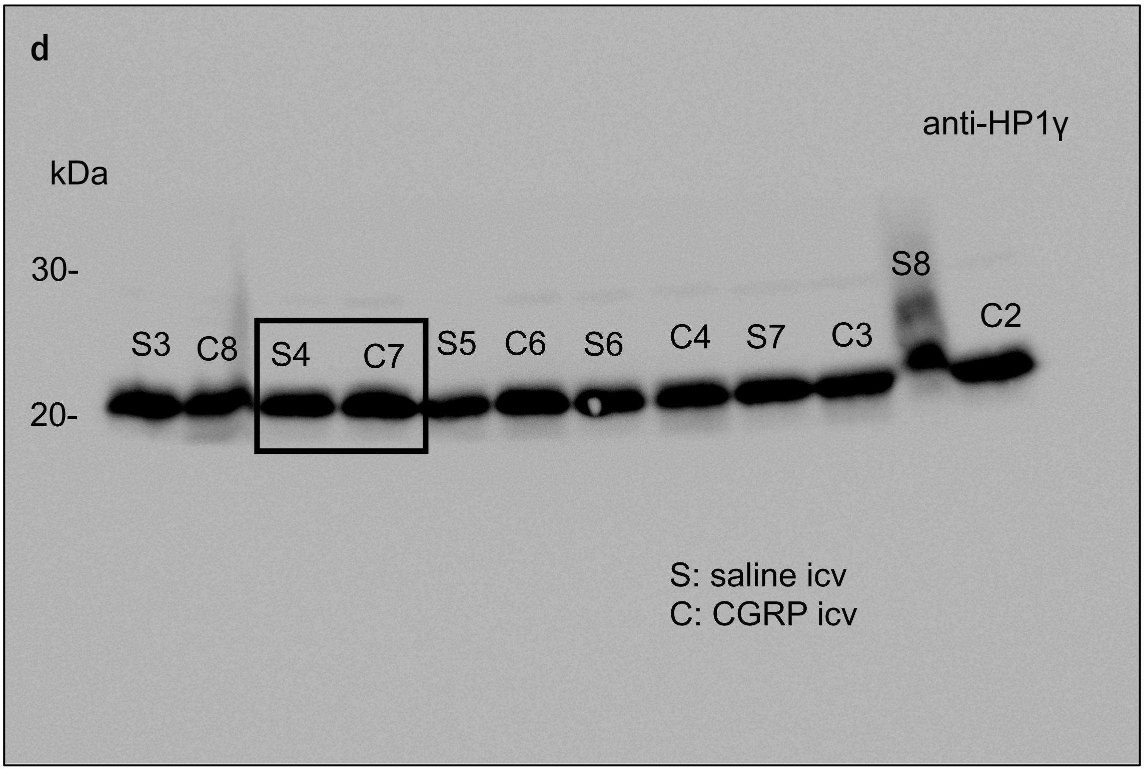


Supplementary Figure 2: Uncropped images of western blots.

(a) Western blot images for Figure 3b, anti-KLF11

(b) Western blot images for Figure 3b, anti-Actin.

(c) Western blot images for Figure 3c, anti-pho-HP1γ.

(d) Western blot images for Figure 3c, anti-HP1γ.

Supplementary Figure 3.

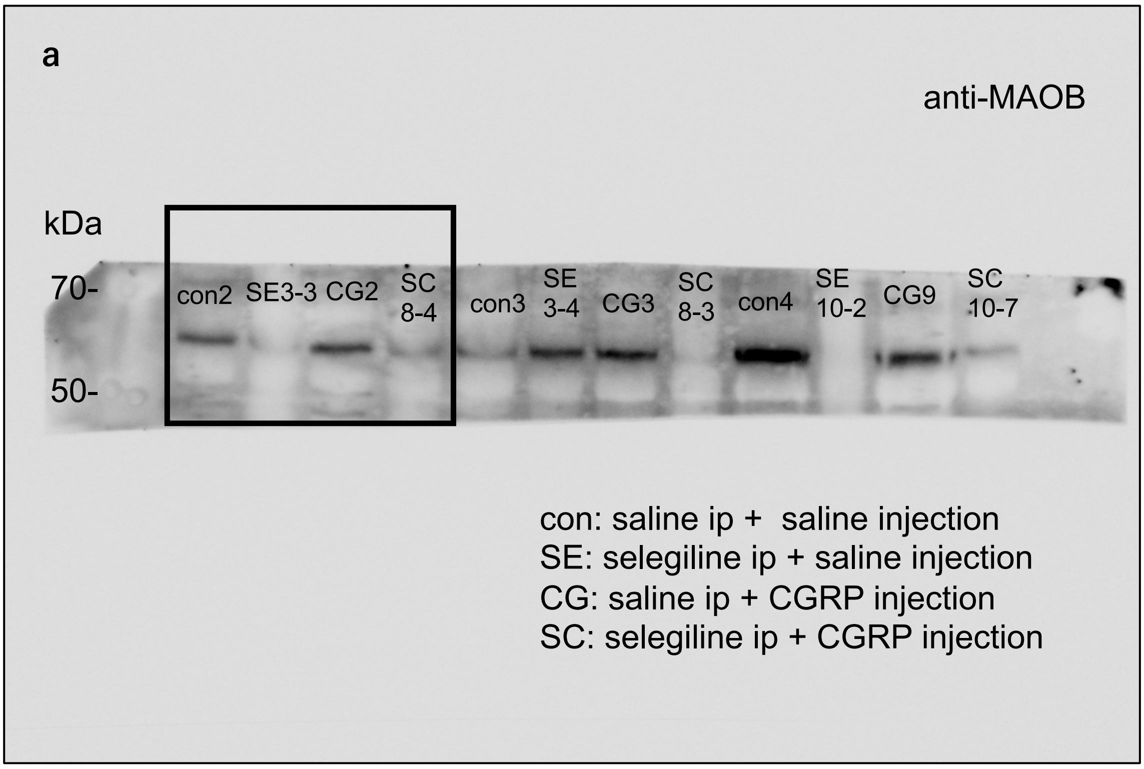


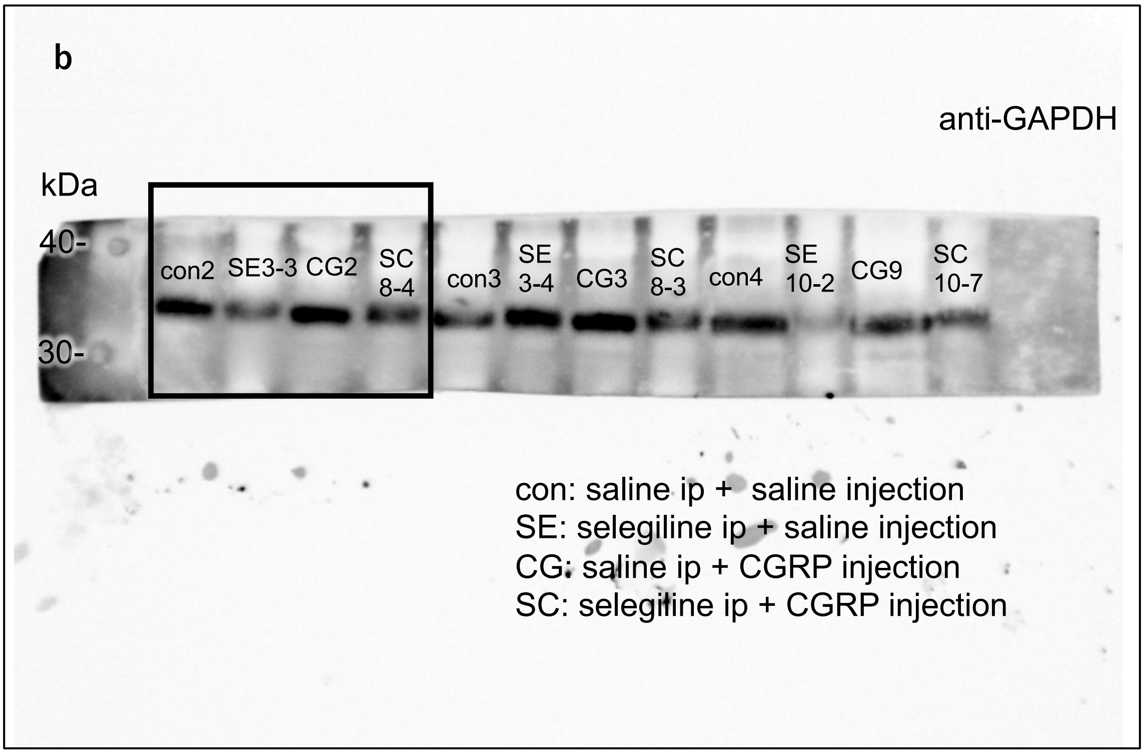


Supplementary Figure 3: Uncropped images of western blots.

(a) Western blot images for Figure 4j, anti-MAOB

(b) Western blot images for Figure 4j, anti-GAPDH.

Supplementary Figure 4.

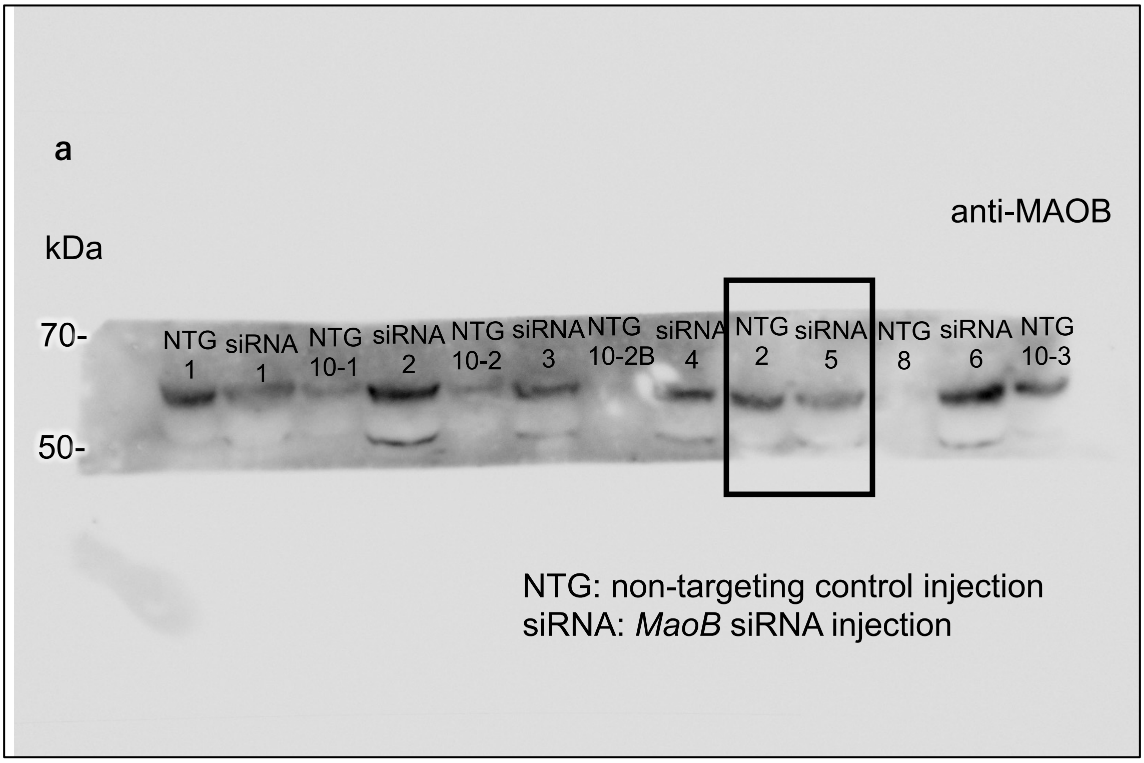


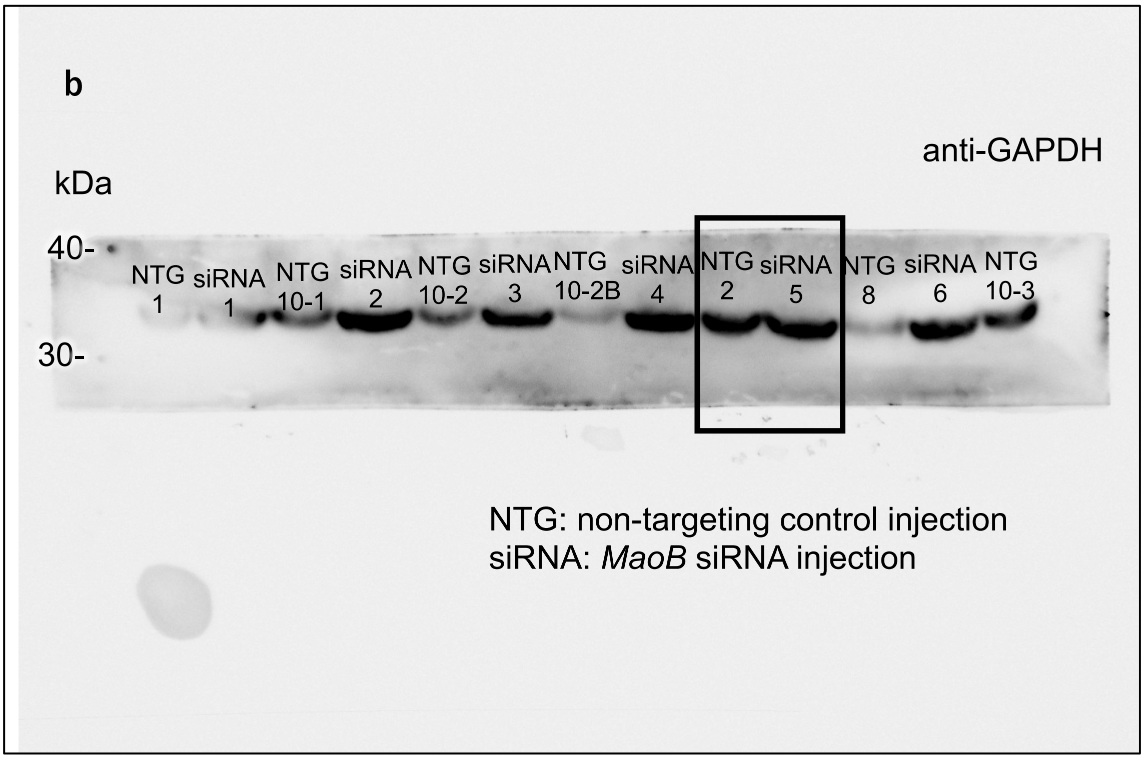


Supplementary Figure 4: Uncropped images of western blots.

(a) Western blot images for Figure 5a, anti-MAOB

(b) Western blot images for Figure 5a, anti-GAPDH.
