# Supplementary material for: CGRP causes anxiety via HP1γ–KLF11–MAOB pathway and dopamine in the dorsal hippocampus
Source: Commun Biol. 2024 Mar 19;7:322. doi: 10.1038/s42003-024-05937-9 (PMC10951359; doi:10.1038/s42003-024-05937-9)
Supplement: Supplementary file 5 — Reporting Summary [file 42003_2024_5937_MOESM5_ESM.pdf]

nature portfolio

Corresponding author(s): Narumi Hashikawa-Hobara

Last updated by author(s): 2024 02 07

Reporting Summary

Nature Portfolio wishes to improve the reproducibility of the work that we publish. This form provides structure for consistency and transparency in reporting. For further information on Nature Portfolio policies, see our Editorial Policies and the Editorial Policy Checklist.

Statistics

For all statistical analyses, confirm that the following items are present in the figure legend, table legend, main text, or Methods section.

n/a	Confirmed	
☐	☒	The exact sample size (n) for each experimental group/condition, given as a discrete number and unit of measurement
☐	☒	A statement on whether measurements were taken from distinct samples or whether the same sample was measured repeatedly
☐	☒	The statistical test(s) used AND whether they are one- or two-sided<br>*Only common tests should be described solely by name; describe more complex techniques in the Methods section.*
☐	☒	A description of all covariates tested
☐	☒	A description of any assumptions or corrections, such as tests of normality and adjustment for multiple comparisons
☐	☒	A full description of the statistical parameters including central tendency (e.g. means) or other basic estimates (e.g. regression coefficient) AND variation (e.g. standard deviation) or associated estimates of uncertainty (e.g. confidence intervals)
☐	☒	For null hypothesis testing, the test statistic (e.g. F, t, r) with confidence intervals, effect sizes, degrees of freedom and P value noted<br>*Give P values as exact values whenever suitable.*
☒	☐	For Bayesian analysis, information on the choice of priors and Markov chain Monte Carlo settings
☒	☐	For hierarchical and complex designs, identification of the appropriate level for tests and full reporting of outcomes
☒	☐	Estimates of effect sizes (e.g. Cohen's d, Pearson's r), indicating how they were calculated

Our web collection on statistics for biologists contains articles on many of the points above.

Software and code

Policy information about availability of computer code

Data collection	N/A
Data analysis	N/A

For manuscripts utilizing custom algorithms or software that are central to the research but not yet described in published literature, software must be made available to editors and reviewers. We strongly encourage code deposition in a community repository (e.g. GitHub). See the Nature Portfolio guidelines for submitting code & software for further information.

Data

Policy information about availability of data

All manuscripts must include a data availability statement. This statement should provide the following information, where applicable:

- Accession codes, unique identifiers, or web links for publicly available datasets
- A description of any restrictions on data availability
- For clinical datasets or third party data, please ensure that the statement adheres to our policy

N/A

April 2023

Research involving human participants, their data, or biological material

Policy information about studies with human participants or human data. See also policy information about sex, gender (identity/presentation), and sexual orientation and race, ethnicity and racism.

Reporting on sex and gender	N/A
Reporting on race, ethnicity, or other socially relevant groupings	N/A
Population characteristics	N/A
Recruitment	N/A
Ethics oversight	N/A

Note that full information on the approval of the study protocol must also be provided in the manuscript.

Field-specific reporting

Please select the one below that is the best fit for your research. If you are not sure, read the appropriate sections before making your selection.

☒ Life sciences ☐ Behavioural & social sciences ☐ Ecological, evolutionary & environmental sciences

For a reference copy of the document with all sections, see nature.com/documents/nr-reporting-summary-flat.pdf

Life sciences study design

All studies must disclose on these points even when the disclosure is negative.

Sample size	approximately 10-15/group
Data exclusions	If the value exceeds 100 times the median, it will be excluded. This is particularly relevant in ChIP assays.
Replication	N/A
Randomization	When the mice were purchased, they were randomly assigned to their own cages.
Blinding	Blinded by two investigator

Reporting for specific materials, systems and methods

We require information from authors about some types of materials, experimental systems and methods used in many studies. Here, indicate whether each material, system or method listed is relevant to your study. If you are not sure if a list item applies to your research, read the appropriate section before selecting a response.

Materials & experimental systems

n/a	Involved in the study	
☐	☒	Antibodies
☒	☐	Eukaryotic cell lines
☒	☐	Palaeontology and archaeology
☐	☒	Animals and other organisms
☒	☐	Clinical data
☒	☐	Dual use research of concern
☒	☐	Plants

Methods

n/a	Involved in the study	
☒	☐	ChIP-seq
☒	☐	Flow cytometry
☒	☐	MRI-based neuroimaging

Antibodies

Antibodies used	mouse monoclonal anti-MAOB (1:1,000; Santa Cruz Biotechnology, Inc., sc-515354),<br>mouse monoclonal anti- TIGE2 (KLF11) (1:1,000; Santa Cruz Biotechnology, sc-136101),<br>rabbit polyclonal anti-p-HP1γ (Ser83) (1:5,000; Invitrogen, Thermo Scientific, PA517210),<br>mouse monoclonal anti-HP1γ (1:5,000; Santa Cruz Biotechnology, sc-398562),

	mouse monoclonal anti-actin (1:10,000; Santa Cruz Biotechnology, sc-8432),<br>horseradish peroxidase-conjugated secondary antibody against mouse or rabbit (1:20,000; Santa Cruz Biotechnology, sc-2357 for mouse, sc-2004 for rabbit)<br>anti HP1γ antibody (1:250 for the hippocampal sample; Santa Cruz Biotechnology),<br>anti-Histone H3 (trimethyl K9) antibody (1:100; Abcam, ab8898)<br>anti-Histone H3 antibody (1:100; Abcam, ab10799)
Validation	Western blotting, ChIP analysis

Animals and other research organisms

Policy information about studies involving animals; ARRIVE guidelines recommended for reporting animal research, and Sex and Gender in Research

Laboratory animals	C57BL6J mice
Wild animals	N/A
Reporting on sex	male
Field-collected samples	N/A
Ethics oversight	Okayama University of Science Animal Care and Use Committee (authorization numbers 2021-002)

Note that full information on the approval of the study protocol must also be provided in the manuscript.

Plants

Seed stocks	N/A
Novel plant genotypes	N/A
Authentication	N/A
